# Epidemiological surveillance of respiratory viral infections in SARS-CoV-2-negative samples during COVID-19 pandemic in Iran

**DOI:** 10.1186/s12985-023-02226-5

**Published:** 2023-12-13

**Authors:** Ali Maleki, Parvaneh Mehrbod, Farah Bokharaei-Salim, Sana Eybpoosh, Mahsa Tavakoli, Azita Eshratkhah Mohammadnejad, Zahra Hosseini, Setareh Kashanian, Laya Farhan Asadi, Mostafa Salehi-Vaziri, Fatemeh Fotouhi

**Affiliations:** 1 COVID-19 National Reference Laboratory, Pasteur Institute of Iran, Tehran, Iran; 2 Department of Influenza and Respiratory Viruses, Pasteur Institute of Iran, Tehran, Iran; 3 Department of Virology, School of Medicine, Iran University of Medical Sciences, Tehran, Iran; 4 Department of Epidemiology and Biostatistics, Research Centre for Emerging and Reemerging Infectious Diseases, Pasteur Institute of Iran, Tehran, Iran; 5 Department of Arboviruses and Viral Hemorrhagic Fevers (National Reference Laboratory), Pasteur Institute of Iran, Tehran, Iran

**Keywords:** COVID-19, SARS-CoV-2, Respiratory viral Infections, Epidemiological surveillance, Syndromic surveillance

## Abstract

**Background:**

To improve the patient care, public health surveillance, and infection control, it is crucial to identify the presence and frequency of the common respiratory infections in individuals with COVID-19 symptoms but tested negative for SARS-CoV-2. This study aimed to shed light on this during the COVID-19 pandemic in Iran.

**Methods:**

In this cross-sectional study, a total of 1,002 patients with acute respiratory infection who had negative SARS-CoV-2 test results and referred to Valfajr Health Center, the National Collaborating Laboratory of Influenza and COVID-19 National Reference Laboratory at Pasteur Institute of Iran were recruited between January 2020 and January 2022. Nasopharyngeal and oropharyngeal swab samples were collected to detect 17 common respiratory viruses via TaqMan one-step real-time multiplex PCR. Demographic and clinical data of the participants were obtained from their electronic medical records.

**Results:**

In total, 218 samples (21.8%) were tested positive for at least one respiratory virus infection. Most of the common investigated respiratory viruses belonged to the years 2020 and 2022. The number of investigated patients in 2021 was few, which highlights the impact of health measures following the COVID-19 pandemic in Iran. Influenza A was the most common virus (5.8%), while adenovirus had the lowest prevalence (0.1%). Although the rate of respiratory virus infection was higher in men (24%) compared to women (19.3%), this difference was not statistically significant (*P* = 0.069). The prevalence of respiratory viruses had an inverse association with increasing age, with the highest rate (55.6%) observed in the age group below 2 years and the lowest rate (12.7%) in those above 65 years.

**Conclusion:**

Our findings underscore the significance of adopting a comprehensive approach to respiratory infections detection and management. These results can be employed for the development of syndromic surveillance systems and implementation of the effective infection control measures. Furthermore, the results contribute to better understanding of the dynamics of respiratory viruses, both during pandemic periods and in non-pandemic contexts.

## Introduction

Severe Acute Respiratory Syndrome Coronavirus 2 (SARS-CoV-2), which belongs to the beta-coronavirus group of *Coronaviridae* family was declared a pandemic by WHO on 11th March 2020 [1]. Like any other respiratory viruses, this virus transmission occurs via contact with infected individuals and respiratory droplets. In the areas with poor ventilation and more than 30 min exposure with infected people, even the airborne transmission is possible [2, 3].

During the pandemic era, the focus of the health centers was on SARS-CoV-2 detection, while the presence of co-infection or other common respiratory infections with or without SARS-CoV-2 has been neglected [4].

The clinical manifestations of SARS-CoV-2 may range from asymptomatic infection (85–90% cases) to mild illness known as influenza like illness (ILI) and severe acute respiratory illness (SARI) requiring hospitalization [5]. They include sore throat, cough, fever, sputum production, fatigue, muscle pain or joint pain, shortness of breath, chills, headache, and other atypical symptoms. These manifestations are not much different from common respiratory viral infections [6]. These similar symptoms of COVID-19 and other respiratory infections can affect the diagnostic and therapeutic efficacy of the COVID-19 suspicious cases. Therefore, identification of the causative respiratory pathogens is of great importance, which contributes to the reduced duration of the patient’s isolation, especially for those who are infected with other common respiratory viruses except for SARS-CoV-2 [7].

A systematic review of the causes of respiratory illness in 2015 confirmed that RSV was the most frequent pathogen detected among patients of different age groups [8]. Later in 2018, a surveillance study on the epidemiology of respiratory illness among hospitalized young children in Chengdu from 2009 to 2014 reported ARI patients with human rhinovirus (HRV) (23.0%), respiratory syncytial virus (RSV) (22.7%), human parainfluenza virus (HPIV) (13.4%), and human bocavirus (HBoV) (8.4%) [9].

Also, during COVID-19 pandemic several studies highlighted decrement in the rate of other respiratory viruses such as RSV [10], influenza A and B, especially B yamagata [11], influenza virus and RSV [12], and influenza A and B RSV, parainfluenza, metapneumovirus, rhino/enterovirus, and seasonal coronaviruses [13].

In this study we aimed to investigate the epidemiological, clinical and virologic characteristics of the suspected COVID-19 but SARS-CoV-2-negative patients, and clarify the infection rate of other respiratory pathogens in Iran during COVID-19 pandemic.

## Materials and methods

### Study design and patients

This cross-sectional study was conducted between January 2020 and January 2022. Totally, 1,002 patients with acute respiratory infections who were referred to the COVID-19 National Reference Laboratory at Pasteur Institute of Iran, the National Collaborating Laboratory of Influenza and COVID-19 National Reference Laboratory at Pasteur Institute of Iran and Valfajr Health Center at Iran University of Medical Sciences, were included. Nasopharyngeal and oropharyngeal swab samples were collected to detect 17 common respiratory viruses via TaqMan one-step real-time multiplex PCR. Demographic and clinical data of the participants were obtained from their electronic medical records.

The eligibility criteria for the patients enrolled in this study were as follows: (i) fever (≥ 37.0 °C) and/or respiratory symptoms (e.g., cough, sputum production, shortness of breath, wheezing, and chest pain, etc.) and (ii) with no other underlying pulmonary diseases. The detailed information, such as demographic and clinical characteristics were recorded in case report forms.

### Viral RNA isolation and sample processing

Nasopharyngeal and oropharyngeal swab samples were collected from the patients. After sampling, the specimens were placed into collection tubes containing 2 mL virus transport media (VTM). Viral nucleic acid was extracted using High Pure Viral Nucleic Acid Kit (Roche, Switzerland) as instructed. Briefly, 200 µl of each sample was mixed thoroughly with the Binding Buffer in the microcentrifuge tube. Following 10 min incubation at room temperature and few steps ethanol precipitating and washing, the pure viral nucleic acid was isolated by adding elution buffer to the column tube and centrifugation. The viral nucleic acid was stored at -80 °C for further analysis.

### SARS-CoV-2 real-time quantitative PCR

SARS-CoV-2 real-time Reverse-transcription PCR (real-time RT-PCR) was performed by 2019-nCoV Nucleic Acid Diagnostic kit (Sansure biotech, Changsha, China) targeting two genomic regions of the virus: open reading frame 1ab (ORF1ab) and nucleocapsid protein (N) according to the manufacturer’s guide. Real-time RT-PCR was done as per the manufacturer’s protocol [14]. Briefly, 30 µL PCR master mix was added to 20 µL RNA template and primers. Thermal cycling was performed at 50 °C for 30 min (for reverse transcription), followed by 95 °C for 1 min (for cDNA pre-denaturation) and 45 cycles of 95 °C for 15 s and 60 °C for 30 s using the Rotor-Gene Q instrument (Qiagen, Germany).

### The multiplex platform for respiratory pathogen panel testing

The extracted RNAs of all SARS-CoV-2-negative samples were tested by three-reaction HiTeq 17 Viro Respiratory Pathogens Multiplex Detection Kit (GeneovA, Iran) for simultaneous identification of 17 respiratory viruses including SARS -CoV − 2, Influenza A, Influenza H1N1, Influenza B, HCoV -HKU1, HCoV -OC43, HCoV -NL63, HCoV − 229E, Metapneumovirus, Respiratory Syncytial Virus, Human Bocavirus 1–3, Human Parainfluenza 1–3, and Adenovirus. The analytical sensitivity and specificity of the tests are 95 and 100%, respectively. Thermal cycling was performed at 50 °C for 15 min (for cDNA synthesis), followed by 94 °C for 3 min (for holding) and 46 cycles of 94 °C for15 s and 60 °C for 30 s on the Rotor-Gene Q instrument (Qiagen, Germany).

### Statistical analysis

Data was analyzed in 3 time periods, before scale-up of the lockdowns (January - March 2020), during lockdowns (March 2020 - March 2021), and after relaxing lockdown measures (March 2021 - Jan 2022) in Iran [15]. Descriptive analyses were performed to characterize the specimens concerning age, gender, underlying disease, and clinical manifestations. Continuous variables were expressed as the mean (± SD) and categorical ones as the number and percentage (%). Logistic regression analysis was used to compare the proportion of respiratory viruses identified in different time periods over the course of the pandemic, as well as in different age groups (toddlers, children and adolescents, adults and elderlies), and diseases outcomes (outpatients, inpatients, and deaths). A P-value of ≤ 0.05 was considered significant. The Stata version 14 software was used for all data analyses.

## Results

### Clinical and demographic characteristics of the cases

Demographic information of the patients (gender, age, pregnancy, underlying diseases, and the outcome of disease) with acute respiratory diseases but COVID-19 negative result participating in the study is shown in Table 1. In total, 1002 individuals presenting with symptoms of acute respiratory infection but tested negative for SARS-CoV-2 were included. The average age was 37.6 years with 520 (51.9%) male and 482 (48.1%) female.

**Table 1:** Demographic information of the patients

Variables		N (%)
**Year of samples**	2020	104 (10.5)
	2021	53 (5.3)
	2022	838 (84.2)
**Sex**	Male	520 (51.9)
	Female	482 (48.1)
**Age** (year) _Mean (SD)_		37.6 (23.3)
**Age Groups**	≤ 2	54 (6.29)
	3–10	63 (7.34)
	11–20	81 (9.44)
	21–30	145 (16.90)
	31–40	192 (22.38)
	41–50	99 (11.54)
	51–60	67 (7.81)
	61–70	58 (6.76)
	71–80	46 (5.36)
	> 81	53 (6.18)
**Pregnancy**	Yes	38 (7.9)
	No	444 (92.1)
**Underlying diseases**	Yes	179 (18.5)
	No	790 (81.5)
**Outcome**	Outpatient	574 (57.3)
	Inpatient	422 (42.1)
	Death	6 (0.6)

### Respiratory viruses identified in SARS-CoV-2-negative patients

A total of 218 samples (21.8%) were tested positive for respiratory viral pathogens (Table 2). The threshold of the test for being positive is Ct ≤ 35. Of these, the highest and the lowest frequencies were related to influenza A virus (58 cases, 5.8%) and adenovirus (1 case, 0.1%), respectively. Seasonal coronaviruses from the genus beta-coronavirus (HCOV-HKU1/OC43) and alpha-coronavirus (HCOV-NL63/229E) ranked second (48 cases, 4.8%) and third (33 cases, 3.5%) in frequency, respectively. In addition, 4 cases (1.8%) of dual co-infections were observed; adenovirus + HCOV-NL63/229E; influenza B virus + RSV, metapneumovirus + HCOV-HKU1/OC43; and parainfluenza virus + HCOV-HKU1/OC43 (Table 2).

**Table 2:** Frequency of respiratory virus infections and dual co-infections

Viral pathogen	Positive	Co-infections	
	N (%)	N = 4	Type
Adenovirus	1 (0.1)	1	HCOV-NL63/229E
Bocavirus (1–3)	5 (0.5)		
Influenza-B	6 (0.6)	1	RSV
RSV	13 (1.3)		
Metapneumovirus	27 (2.7)	1	HCOV-HKU1/OC43
Parainfluenza virus (1–3)	27 (2.7)	1	HCOV-HKU1/OC43
HCOV-NL63/229E	33 (3.5)		
HCOV-HKU1/OC43	48 (4.8)		
**Influenza-A**	**58 (5.8)**		
Negative	784 (78.2)		

### Frequency and prevalence of respiratory virus Infections according to age and gender

**Fig. 1:**
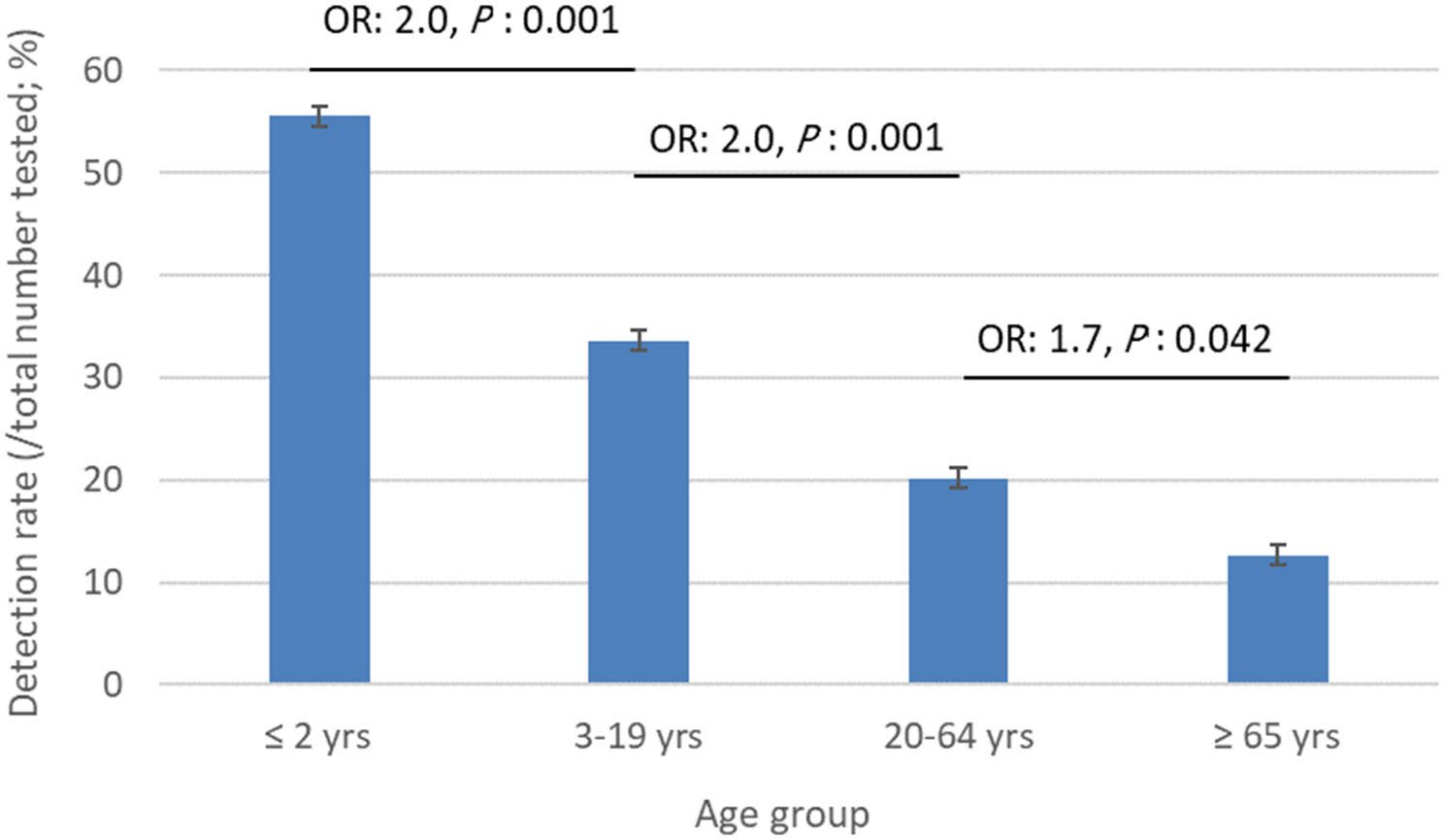
Frequencies of respiratory virus infections in different age groups. With increasing age, the frequency of common respiratory infections in people suspected with COVID-19 but negative PCR test decreased significantly

The frequency of respiratory virus infections in different age groups was shown in Fig. 1.

As can be seen in the Figure, there is a significant difference in the frequency of respiratory virus infection among age groups, and the infection rate decreased generally with increasing age (*P* < 0.05). The highest rate of confirmed viral infection (55.6%) detected by qPCR was observed in children 2 years old and younger, and the chance of infection in this age group was 2 times higher compared to the patients aged 3 to 19 years. The chance of getting respiratory virus infections in the age group of 3 to 19 years was determined twice as compared to the next age group of 20 to 64 years old. Similarly, the chance of infection in the age group of 20 to 64 years was higher than people over 65 years old (1.7 times).

**Fig. 2:**
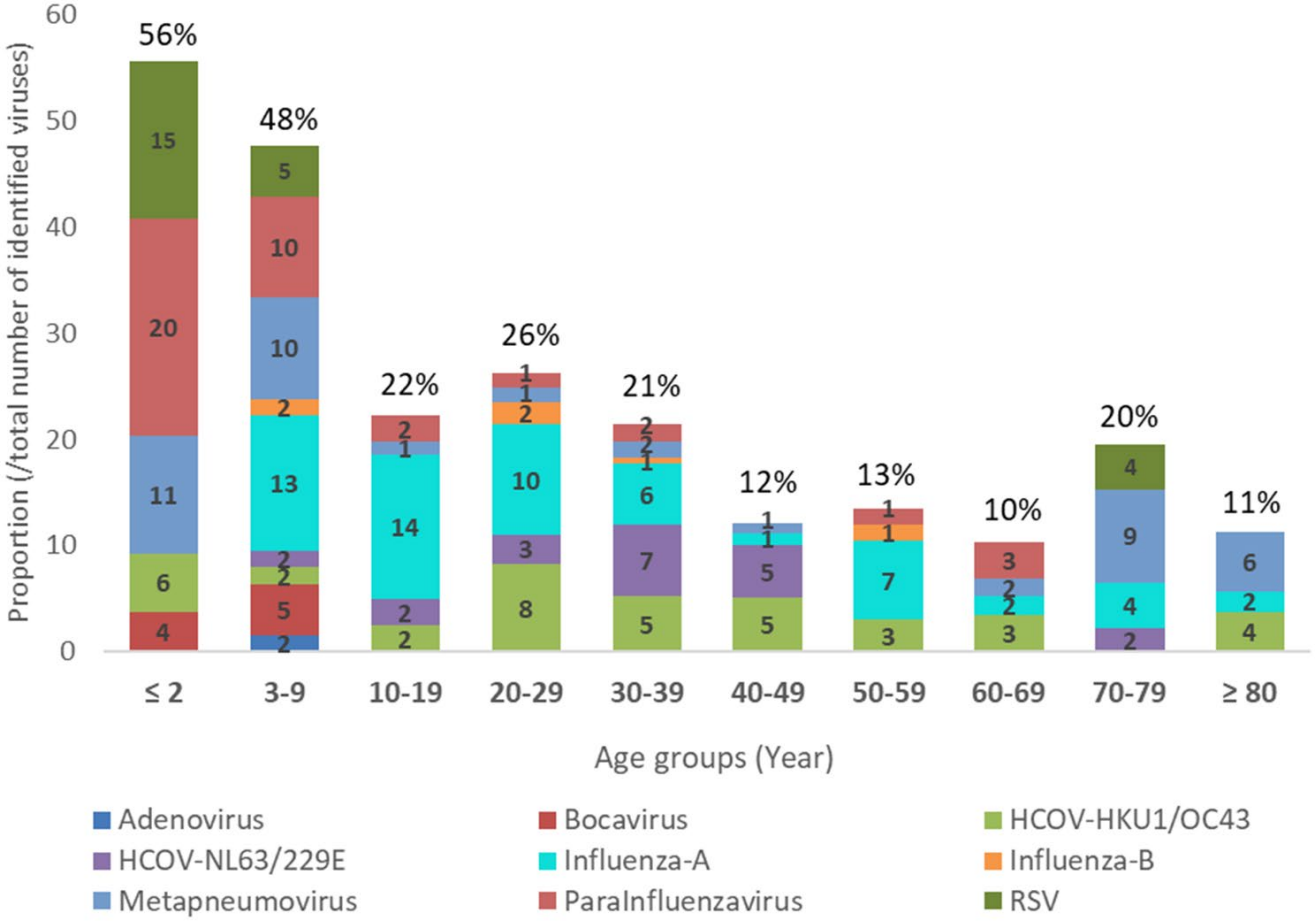
Detection rate and identification of respiratory viral pathogens in COVID-19 suspected samples with negative SARS-CoV-2 PCR test result in different age groups. The highest prevalence of common respiratory infections was observed in children bellow 10 years. The values inside the graphs show the number of each respiratory virus within each group to the total number of samples evaluated in that group. The value above each bar represents the total prevalence of respiratory viruses (regardless of the type) in each age group

The distribution of infection based on the type of the virus in the age groups was also significant, so that metapneumovirus, parainfluenza virus, and RSV were more frequently observed in children (younger than 10 years old) and elderly people (over 65 years old), while influenza viruses B and HCOV-HKU1/OC43 were more prevalent in people between 20 and 65 years old (Fig. 2).

No significant difference was observed in the frequency of respiratory virus infections based on the gender (*P* = 0.069), although this rate was slightly higher among men (24%) than women (19.3%).

### The association of virus and severity of the infection

**Fig. 3:**
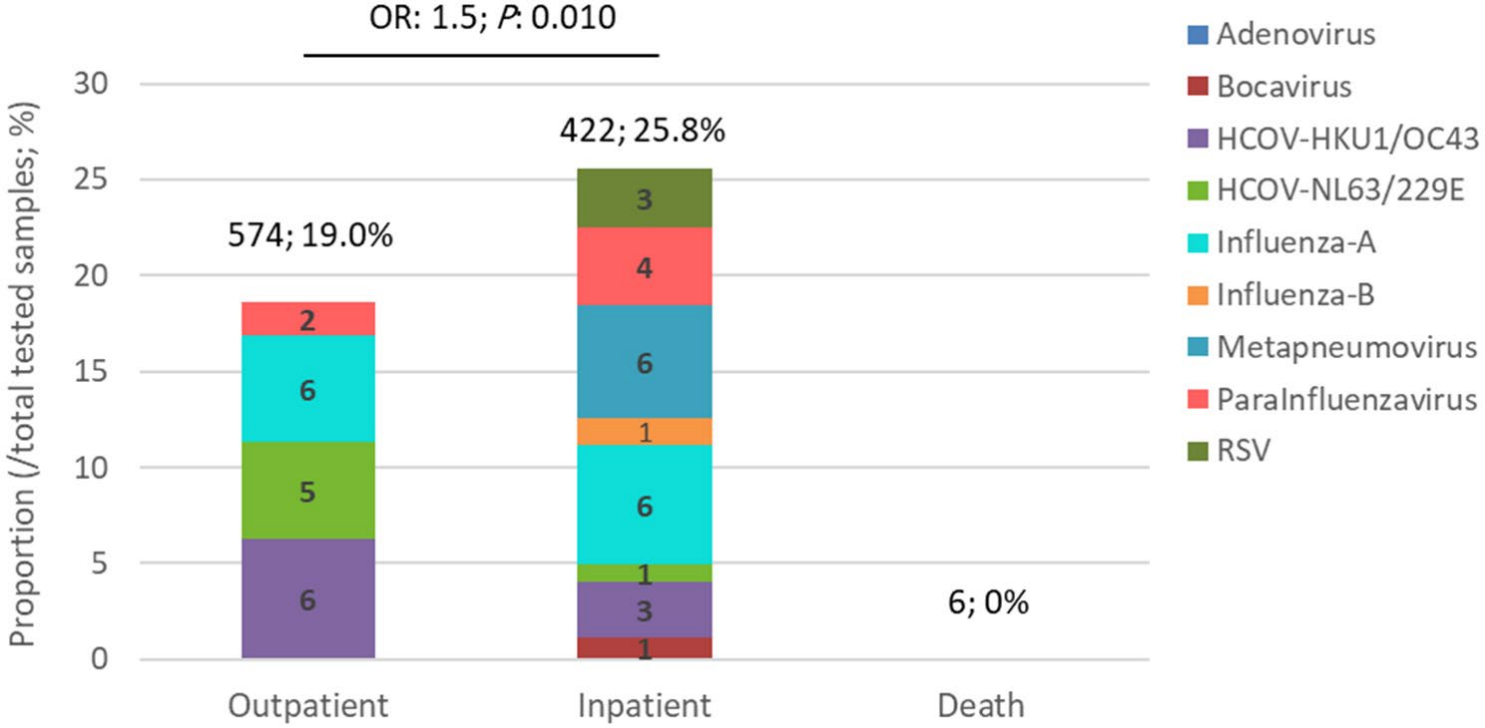
Detection rate and identification of respiratory viral pathogens in COVID-19 suspected samples with negative SARS-CoV-2 PCR test result based on the disease severity. The values above the bars indicate the total number of samples evaluated in each group and the percentage of the total viruses identified (excluding virus type) in that group. The values inside the bars show the percentage of each virus in that group. In the suspected deceased persons (6 people), no case of the desired viruses was detected. The chance of isolation of the desired viruses in the hospitalized patients was 1.5 times higher than that of outpatients. This difference was statistically significant (*P* = 0.010)

A significant association was observed between respiratory virus infection and disease severity. The rate of infection in the hospitalized patients (25.8%) was higher than outpatients (19%) (*P* = 0.010). In addition, the variety of viral infections was higher in the hospitalized patients compared to the outpatients (9 different viruses versus 5, respectively). None of the viruses included in the panel was detected in the 6 death cases (Fig. 3).

### Frequency and prevalence of respiratory virus Infections in different time periods of the COVID-19 pandemic

**Fig. 4:**
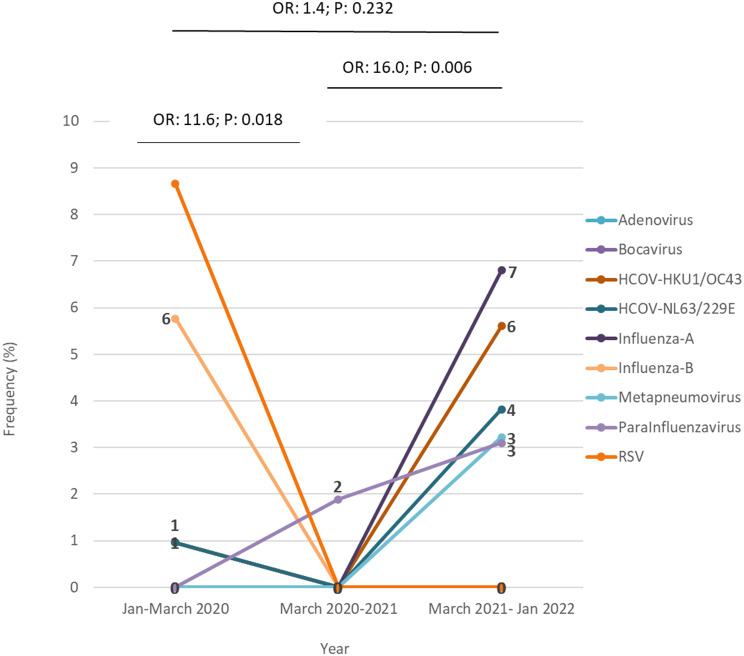
Detection rate and identification of respiratory viral pathogens in COVID-19 suspected samples with negative SARS-CoV-2 PCR test result in different time periods over the course of the pandemic, 2020-2022. The values above the bars indicate the total number of the samples evaluated and the percentage of the total viruses detected in each year (excluding virus type). The values inside the bars indicate the percentage of each virus in that group. In 2021, in only 2% of total 53 negative samples just parainfluenza virus was detected. The variety of respiratory viruses identified in 2022 was the highest (23.5%). The difference in the frequency of detected viruses before and after the peak period of COVID-19 compared to the peak period of COVID-19 (2019–2020) was significant, but before and after the peak of COVID-19, no significant difference was observed in the prevalence of infections (*P*˃0.05)

Most of the investigated samples belonged to the years 2020 and 2022. The number of investigated patients in 2021 was few (53 cases), which was related to the impact of health measures following the COVID-19 pandemic in Iran. As seen in Fig. 4, the distribution of respiratory viruses in 2022 was different compared to the beginning of the pandemic, such that in 2020, RSV and influenza B viruses were dominant, while influenza A viruses and seasonal coronaviruses had more circulation in 2022. The values displayed above the bars represent the total number of samples examined in each group and the percentage of identified viruses (excluding virus type) within that group.

**Fig. 5:**
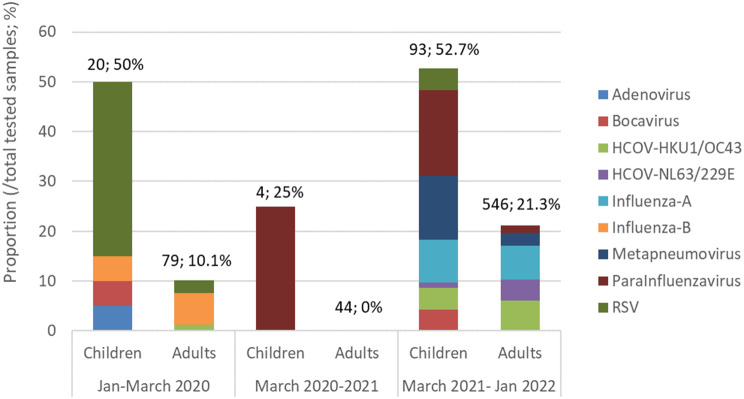
Detection rate and identification of respiratory viral pathogens in SARS-CoV-2-negative children (≤ 10 years) and adults (≥ 20 years) in Iran, 2020–2022. The values above the bars indicate the total number of samples evaluated in each group and the percentage of the total viruses identified (excluding virus type) in that group. The values inside the bars show the percentage of each virus in that group. In the adults group in 2021 (44 individuals), no viruses were detected. The difference in the frequency of detected viruses between children and adults were significant in different time periods, with children exhibiting significantly higher detection rates and diversity of detected viral respiratory viruses (*P* < 0.0001). Detection rate of respiratory viruses during the pandemic decreased significantly in both age groups when compared to before and after the pandemic. Before and after the pandemic, the detection rate remained relatively the same in both groups; however, there were changes in the distribution of infecting viruses

Focusing on age groups, we detected no respiratory viruses in the adult group (≥ 20 years) during 2021 (consisting of 44 individuals). There was a significant difference in the frequency of detected viruses between children and adults during different time periods, with children exhibiting significantly higher detection rates and a greater diversity of viral respiratory viruses (*P* < 0.0001). The confirmed detection rate of viral respiratory pathogens detected by qPCR decreased significantly in both age groups during the pandemic compared to before and after it. However, the detection rate remained relatively consistent in both groups before and after the pandemic. Notably, there were changes in the distribution of infecting viruses (Fig. 5).

## Discussion

The most common causes of various infections in the lungs and respiratory system are respiratory viruses; moreover, their prevalence through the year varies in different seasons, which is conducted by the retrospective epidemiologic studies. Since the beginning of the COVID-19 pandemic in the early 2020, the non-SARS-CoV-2 respiratory infections decreased dramatically, which resulted in a significant drop in the diagnosis of such viruses mentioned in several publications like RSV [10], influenza B, that its extinction was suggested [11], RSV and influenza virus together [12], and also common respiratory viruses such as influenza A and B viruses, RSV, parainfluenza, metapneumovirus, rhino/enterovirus, and seasonal coronaviruses [13]. The global dominance of SARS-CoV-2 infection, and restrictions applied against COVID-19 such as quarantines, social distancing, virtual gatherings, and hygiene instructions, repetitive hand washings, high-level surface disinfections, and wearing masks led to the alterations in the pattern of other respiratory viruses distribution all over the world [16].

In the second year of pandemic, the governments started to reduce the restriction guidelines gradually; therefore, it resulted in the growth of other viral respiratory infection cases by the following year [17].

In this study, the oropharyngeal/nasopharyngeal swab samples of the patients with respiratory symptoms but negative COVID-19 PCR test results, were assessed for other 17 respiratory viruses. Most of the results of this study belong to the year 2020 and 2022, which corresponds to the time of legal restrictions reduction all over the world, including Iran. The gender composition and the percentage of inpatient-outpatient cases were chosen relatively equal to reduce the analytical error. The positivity of 28 samples (21.8%) for at least one viral infection other than SARS-CoV-2 in the period of our sampling confirms the fact that even in a viral pandemic, the rest of the viruses are still able to circulate and cause disease.

It also indicates that the majority of the studied population (about 80%) in our study got sick with other infectious agents including the rest of the viruses other than SARS-CoV-2 and bacteria. The number of viral infection cases is generally predominant compared to the bacterial ones with symptoms such as sepsis, sore throat, and yellowish sputum [18, 19], but the incidence of bacterial secondary infection as super-infection is also very common. Therefore, detection of causative agents of the remaining 80% of the cases is very important, which might include high percentages of other infections that were not detected in our study such as common rhinoviruses that was not included in our detection kit panel.

Based on our study, among the samples with at least one viral infection, the highest rate was allocated to influenza A virus, which is in accordance with other studies [18, 19]. It was followed by the seasonal coronaviruses [20, 21], and the least infection was developed by adenoviruses which was in contrast to the results before the COVID-19 outbreak over the world [19, 22–24].

Generally, most of the cases were young infants age < 2y (approx. 55%), which was predictable based on their immune system evolution [25]. Although, it was expected to have the same frequency with the elderly adults age > 65y according to their vulnerability, underlying diseases, and insufficient immune responses; the least number of the patients were identified in this category: however, it must be noticed that most of the consequential preventive guidelines were applied to elderly people during the pandemic period; in addition, an effective healthcare services were applied to them even when the signs were not much life-threatening.

The significant increase in the hospitalized patients compared to the outpatients is also of importance. This is debatable that either these types of viral infections caused patients’ hospitalization, or hospitalization made the patients expose with various viral agents. However, the latter might be supported by the viral diversity (9 types) among the hospitalized participants.

Our study implicitly confirmed the proper and reliable function of the virological laboratories. It was previously undervalued by the public or some physicians that claimed the lack of precision and profession in the laboratories in addition to COVID-19 PCR test inefficiency caused the negative results of the patients with severe symptoms in the outbreak. Although the functionality and reliability of some laboratories and tests could be criticized in general but the study revealed that even in the middle of a pandemic other non-related infectious agents may have the ability to circulate and cause infection or disease as well.

The results of this study underline the importance of using molecular techniques with the ability to simultaneously detect multiple infectious agents (such as multiplex qRT-PCR) for syndromic surveillance of respiratory viral infections. The method used in this study, in addition to rapid detection of various viral agents, has also provided the possibility of detecting simultaneous infections (co-infection) of two or more viruses [21, 26].

It is necessary to mention the constraints of the study. Small sample size in some periods of time due to COVID-19 preventive measures, and the lack of proper access to the respiratory samples are the most limitations of our study. The results would have been more reliable and meaningful if samples could be collected weekly over different years, but due to access limit and economic constraints, this was not feasible to conduct. Also, one of the other important limitations of this study was the inability of the used diagnostic kit to detect rhinoviruses, while rhinoviruses are the most common cause of common cold through the population annually.

## Conclusion

In this study, the prevalence of respiratory viruses other than SARS-CoV-2 was investigated during the COVID-19 pandemic. This is one of the first studies investigated simultaneous detection during SARS-CoV-2 infection in Iran. In this period of time, influenza A virus and adenovirus showed the highest and the lowest prevalence, respectively. By increasing the age, the prevalence of respiratory viruses decreased mostly due to the preventive measures applied to the elder population more strictly. During the peak of SARS-CoV-2 prevalence (March 2020-March 2021), due to applying the quarantine, travel restriction, face mask, etc., the only common viral pathogen detected in our study was parainfluenza virus. Simultaneous screening of common viral respiratory pathogens will help to expand our understanding about the epidemiology and dynamics of common viral types during pandemics and also help the clinicians to diagnose the exact viral disease to assist patients both during pandemic periods and in non-pandemic contexts.

## Data Availability

All data are available in case of need.
